# A Chemist with a Strange Etiology of Rhabdomyolysis: A Case Report of a Rare Toxicological Emergency

**DOI:** 10.5811/cpcem.2021.7.52923

**Published:** 2021-09-09

**Authors:** Rajadurai Meenakshisundaram, Joshua Vijay Joseph, Prabakaran Perumal, Akmal Areeb, Prathap Pancheti, Dinesh Kannan Sampath, Esther Monica Jared

**Affiliations:** Apollo KH Hospital, Department of Emergency Medicine and Critical Care, Melvisharam, Tamil Nadu, India

**Keywords:** chloroform, poisoning, rhabdomyolysis, N-acetylcysteine

## Abstract

**Introduction:**

Chloroform, a halogenated hydrocarbon, causes central nervous depression, hepatotoxicity, nephrotoxicity, and rhabdomyolysis. Historically, chloroform had been used as a general anaesthetic and today is still used in chemical industries. Lack of proper personal protective equipment and adequate knowledge about its toxic effects can lead to serious harm.

**Case report:**

A 33-year-old gentleman presented to the emergency department (ED) with altered mental status. Given his depressed mental status, the decision was made to intubate shortly after arrival for airway protection. Further history raised suspicion of occupational chloroform exposure. Brown-colored urine further strengthened suspicion of chloroform poisoning with resultant rhabdomyolysis. Forced alkaline diuresis and N-acetylcysteine were started in the ED. His mental status and respiratory efforts improved on hospital day two, and he was ultimately extubated. Creatine phosphokinase and myoglobin levels were initially high but gradually came down by hospital day six. On hospital day 10, the patient was deemed stable and safely discharged.

**Conclusion:**

A patient with chloroform inhalation who suffered resultant rhabdomyolysis and hepatotoxicity was successfully treated with early initiation of forced alkaline diuresis, N-acetylysteine, and hemodialysis.

## INTRODUCTION

Chloroform, or trichloromethane (CHCl_3_), is a colorless, volatile liquid with a pleasant ethereal odor. Although no longer used as an anaesthetic, chloroform is still used in industrial applications as an intermediate in chemical syntheses. Available human data on acute chloroform exposure are from older studies that tested various exposure regimens (680–7200 parts per million for 3–30 minutes); effects reported included detection of strong odor, headaches, dizziness, and vertigo.^1^ We describe an early identification and successful outcome of inadvertent chloroform inhalation in a chemist with rhabdomyolysis and hepatic injury.

## CASE REPORT

A 33-year-old gentleman presented to the emergency department (ED) with altered mental status. Given his depressed mental status, the decision was made to intubate shortly after arrival for airway protection. Further history revealed that the patient had been working overnight in a chemical factory and returned home in the morning. Since his return, he had complaints of headache and giddiness. After about two hours, he began vomiting and then became unconscious. A focused history was obtained from relatives regarding the possibility of drug overdose or poisoning. Later in his evaluation, the medical team was able to contact a colleague from the chemical factory who revealed the patient had been working with high density chloroform all night in his role as a senior scientist with expertise in re-refining lubricants and oils, as well as reverse engineering of customized products. This raised suspicion of occupational high-density chloroform exposure as the cause of the patient’s presentation. Brown-colored urine further strengthened the suspicion of chloroform poisoning with resultant rhabdomyolysis. Forced alkaline diuresis (FAD) was initiated within an hour of arrival to the ED, as is customary at our institution, via a multidisciplinary approach with the nephrology and internal medicine services.

The urine alkalanisation by FAD was performed as follows: 500 millilitres (mL) 0.9% normal saline over one hour(hr), followed by 50 milliequivalents (mEq) sodium bicarbonate in 5% dextrose 500 mL over one hr, 20 mEq potassium chloride in 500 mL 0.9% normal saline over one hr, followed by furosemide 40 milligrams (mg) intravenous (IV). This cycle was repeated for the next five days with a target urine pH above 8. The patient was also treated with IV-administered N-acetylcysteine (NAC) 150 mg per kilogram (kg) over one hr, followed by 50 mg/kg over 4 hr, and then was started on an IV infusion at a rate of 6.25 mg/kg/hr. Early dialysis was planned for renal protection and to remove dialyzable compounds; two cycles of ultrafiltration were given on consecutive days. His mental status and respiratory efforts improved on hospital day two, and he was able to be extubated. He confirmed the occupational exposure to high-density chloroform while working in the laboratory, as well as his symptoms of headache and giddiness since then.

His blood work was significant for elevated transaminases and hyperbilirubinemia (Table). Other significant events of his hospital course included QTc interval prolongation noted on hospital day two, without any accompanying cardiac dysrhythmias. Creatine phosphokinase and myoglobin levels were initially high but downtrended by hospital day six. After 10 days in the hospital the patient was able to be safely discharged home. While measurement of blood levels of chloroform was considered, this test was not available at his treatment facility. On follow-up, the patient was clinically asymptomatic and all investigations were within normal limits. The patient was counseled about proper protective equipment for the workplace to avoid further dangerous occupational exposure. The timeline of management is shown in Figure.

## DISCUSSION

In the past, chloroform was used as an inhalational anaesthetic. Today it is used in chemical industries.^1^ Chloroform toxicity in the modern day is relatively uncommon; the most recent literature is from about 30 years ago. The most common way that humans are exposed to chloroform is through inhalation; however, poisonings through ingestion have also been recorded.^2^ When chloroform was used as an inhalational anaesthetic, the delayed effects observed included drowsiness, restlessness, vomiting, fever, tachycardia, jaundice, liver enlargement, abdominal tenderness, abnormal liver and kidney function, delirium, and coma. Chloroform may increase cardiac sensitivity to epinephrine, increasing the risk for arrhythmias. Some studies have found that chloroform may also cause injury to red cell membranes, clotting defects, and rhabdomyolysis.^3^

**CPC-EM Capsule**

What do we already know about this clinical entity?

*Rhabdomyolysis secondary to toxins is managed by forced alkaline diuresis to prevent renal failure.*

What makes this presentation of disease reportable?

*Rhabdomyolysis is a rare entity presenting to the emergency department after inhalation of chloroform.*

What is the major learning point?

*Occupational exposure should be considered in patients presenting with unknown poison. Timely management of rhabdomyolysis and chloroform toxicity will improve outcomes.*

How might this improve emergency medicine practice?

*Early recognition and initiation of management based on occupational exposure should be considered in the emergency department..*

Cytochrome P450 2E1-mediated oxidation plays a major role in chloroform toxicity.^4^ The utilization of NAC for chloroform-induced hepatotoxicity has demonstrated successful outcomes in cases with mild hepatotoxicity. The pathophysiology behind this is thought to be that as chloroform causes hepatic damage through free radical injury, NAC may combat this my repleting glutathione and scavenging free radicals, ultimately decreasing hepatic injury secondary to chloroform exposure.^5^

Rhabdomyolysis is a rapid dissolution of skeletal muscle, which leads to release of electrolytes and intracellular muscle components (such as myoglobin, creatine phosphokinase, aldolase, and lactate dehydrogenase) into the bloodstream and extracellular space. Fluid repletion is important to prevent prerenal azotemia. This repletion is provided with 500 mL/hr saline solution alternated every hour with 500 mL/hr of 5% glucose solution with 50 millimoles of sodium bicarbonate for each subsequent 2-3 L of solution. A urinary output goal of 200 mL/hr, urine pH greater than 6.5, and plasma pH greater than 7.5 are reasonable targets.^6^

## CONCLUSION

This case report describes a young man who was found to have rhabdomyolysis and hepatic injury after he presented with an uncommon chemical poisoning. He was successfully managed with general measures, N-acetylcysteine administration, forced alkaline diuresis, and dialysis support. High clinical suspicion and focussed history based on occupational exposure were helpful in identifying the compound and initiating appropriate early management.

## Figures and Tables

**Figure:**
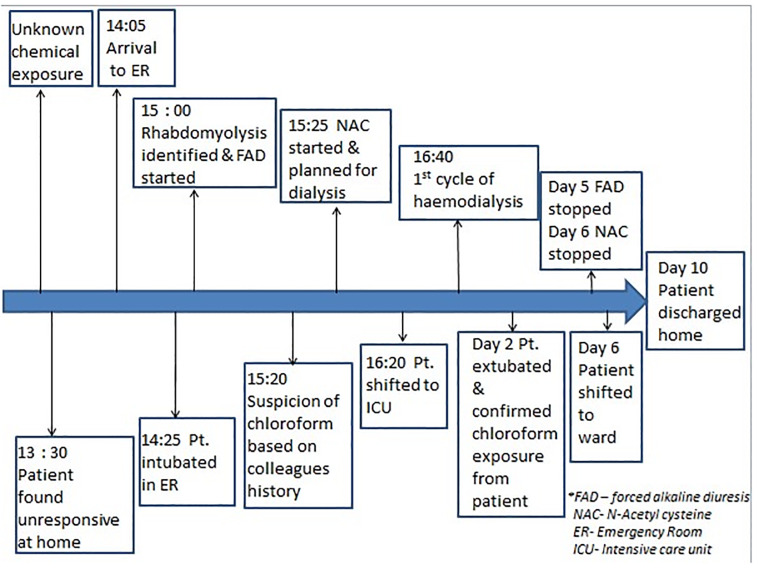
Timeline of our management of patient with chloroform toxicity. *ER*, emergency room; *Pt*, patient; *NAC*, N-acetyl cysteine; *ICU*, intensive care unit; *FAD*, forced alkaline diuresis.

**Table:** Laboratory parameters in a patient with chloroform toxicity.

Day of admission	Normal range	1^st^	3^rd^	5^th^	8^th^	10^th^ (discharged)	Follow-up (4 weeks)
Haemoglobin (g/dL)	12–15	13.8	14.0	13.8	12.2	12.6	12.2
Total counts (cells/mm^3^)	4000–11000	15260	16780	14700	11020	10640	8100
Platelet count (cells/mm^3^)	150000–450000	324000	246000	208000	228000	258000	394000
Urea (mg/dL)	17–43	17	17	14	23	20	13
Creatinine (mg/dL)	0.7–1.2	1.2	1.1	0.9	1.1	1.1	0.8
Total bilirubin (mg/dL)	0.1–1.2	0.6	3.4	6.2	3.8	2.2	0.4
Direct/Indirect (mg/dL)	0.2/0.4	0.2/0.4	0.8/2.6	2.7/3.5	2.5/1.3	1.2/1.0	0.1/0.3
SGOT/SGPT (IU/L)	10–50/10–50	39/28	426/104	398/688	204/599	108/298	76/102
Sodium/ Potassium (mEq/L)	135–145/ 3.5–5	139/4.3	135/3.0	132/3.7	128/3.2	132/3.4	138/3.2
Serum Calcium (mg/dL)	9–11	7.9	7.5	7.5	8.5	8.6	8.8
Serum Phosphorus (mg/dL)	3–4.5	1.9	1.4	2.4	2.2	2.4	2.8
PT/INR (sec)	13–15/1	15/1	27/1.9	24/1.67	20/1.3	20/1.1	17/1.1
CPK (IU/L)	0–195	20390	34040	11830	1500	634	184
Serum myoglobin (ng/mL)	25–80	5895	-	517	214	-	-
Urine myoglobin (ng/mL)	<5	2625	-	-	-	-	-
LDH (IU/L)	140–280	480	1118	605	273	-	128
ECG QTc interval (ms)	431–450	413	511	466	423	415	410

*SGOT*, serum glutamic oxaloacetic transaminase; *SGPT*, serum glutamic pyruvic transaminase; *PT,* prothrombin time; *INR,* international normalised ratio; *CPK,* creatine phosphokinase; *LDH,* lactate dehydrogenase; *ECG,* electrocardiography; *g/dL*, grams per decilitre; *mm^3^*, cubic milimetres; *mg/dL*, milligrams per decilitre; *ng/mL*, nanograms per millilitre; *IU/L*, international units per litre; *ms*, milliseconds.
